# Successful palliation for an aged patient with primary pericardial mesothelioma

**DOI:** 10.1186/s12957-015-0692-5

**Published:** 2015-09-17

**Authors:** Ryutaro Isoda, Hiromichi Yamane, Shintaro Nezuo, Yasumasa Monobe, Nobuaki Ochi, Yoshihiro Honda, Satoshi Nishimura, Maki Akiyama, Takeshi Horio, Nagio Takigawa

**Affiliations:** Clinical Education and Training Center, Kawasaki Hospital, Kawasaki Medical School, 2-1-80 Nakasange, Okayama, 700-8505 Japan; Department of General Internal Medicine 4, Kawasaki Medical School, 2-1-80 Nakasange, Okayama, 700-8505 Japan; Department of General Internal Medicine 3, Kawasaki Medical School, 2-1-80 Nakasange, Okayama, 700-8505 Japan; Department of Pathology 1, Kawasaki Medical School, 2-1-80 Nakasange, Okayama, 700-8505 Japan

**Keywords:** Primary pericardial mesothelioma, Aged patient, Pericardiocentesis, Supportive care

## Abstract

An 85-year-old Japanese man with a complaint of exertional dyspnea was admitted to our hospital. Sixty-three years prior to admission at our hospital, he handled asbestos for 2 years in a factory. His chest computed tomography showed a massive pericardial effusion leading to cardiac tamponade and right pleural plaque. After a pericardiocentesis was performed, he recovered from cardiac failure caused by the cardiac tamponade. Pathological examination of the pericardial effusion revealed malignant mesothelial cells. Therefore, he was diagnosed with primary pericardial mesothelioma (PPM) related to asbestos exposure. Although his disease slowly progressed over 18 months, he remained active without any adjuvant treatments such as chemotherapy. Long-term palliation in an aged patient with PPM is rarely obtained using supportive care alone because the prognosis of PPM has been consistently reported to be very poor and almost fatal within a year. Clinical oncologists and thoracic surgeons should be aware of this disease because the accumulation of knowledge on PPM may lead to successful treatment even in aged patients.

**Electronic supplementary material:**

The online version of this article (doi:10.1186/s12957-015-0692-5) contains supplementary material, which is available to authorized users.

## Background

Primary pericardial mesothelioma (PPM) is an extremely rare malignant neoplasm that arises from the pericardial mesothelial cell layers [1]. The incidence of PPM has been reported to be lower than 0.0022 % on an autopsy series [2]. The prognosis of PPM, which is usually unresectable, is very poor and almost consistently fatal [3]. Although no standard treatment has been established for this disease entity, subxiphoid pericardiostomy followed by drainage may be a safe procedure to provide effective and durable symptomatic relief in critically ill patients [3]. Here, we report an unusual clinical case, wherein a pericardiocentesis led to long-term palliation in a patient with PPM.

## Case presentation

**Fig. 1:**
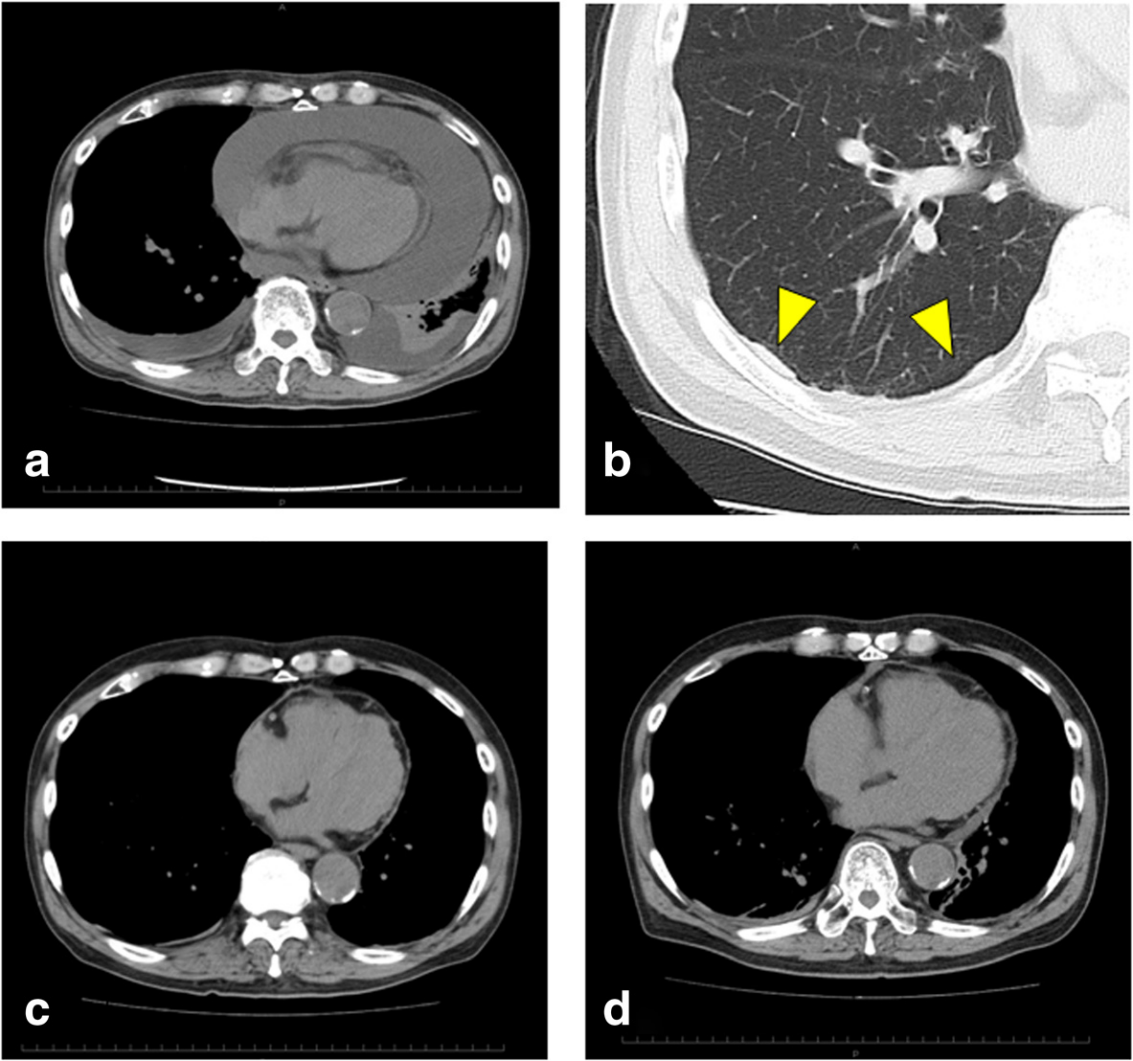
Findings from chest computed tomography. A chest computed tomography (CT) revealed massive pericardial effusion and bilateral pleural effusions on admission (**a**). A chest CT after 8 months clearly showed pleural plaques (**b** *yellow arrow heads*). A chest CT revealed that the pleural effusion was well-controlled for 8 months (**c**) and 16 months (**d**) after the initial treatment. The maximum thicknesses of the cavity in which the pericardial effusion accumulated in Fig. 1a, c, and d were 34.8, 3.3, and 12.6 mm, respectively

An 85-year-old man with 30 pack-year history of smoking presented to our hospital with a complaint of chest discomfort and exertional dyspnea persisting since a month. He had suffered myocardial infarction 9 years prior to this episode. He had worked as a gardener for about 40 years. Prior to becoming a gardener, he handled asbestos for 2 years in a factory. An electrocardiogram on admission revealed sinus tachycardia of 110 beats per minute. We observed a mild deterioration of his oxygen saturation to 93 % on room air with effort. His chest computed tomography (CT) revealed a massive pericardial effusion and bilateral mild pleural effusions without any tumors; these findings likely eliminated the diagnoses of primary lung cancer and metastatic carcinomas (Fig. 1a). In addition, right pleural plaques were clearly observed on chest CT after 8 months (Fig. 1b and Additional file 1: Figure S1). Cardiac ultrasonography also showed a massive pericardial effusion that caused a cardiac tamponade. A needle pericardiocentesis was immediately performed. The total amount of removed pericardial fluid was 2300 mL. This fluid was a bloody exudate with a specific gravity of 1.032. The chest discomfort and dyspnea markedly improved after the pericardiocentesis. To maintain the improvements in his symptoms, we initiated the administration of loop diuretics (20 mg/day). No further drainage or invasive approaches were performed to control the pericardial effusion.

**Fig. 2:**
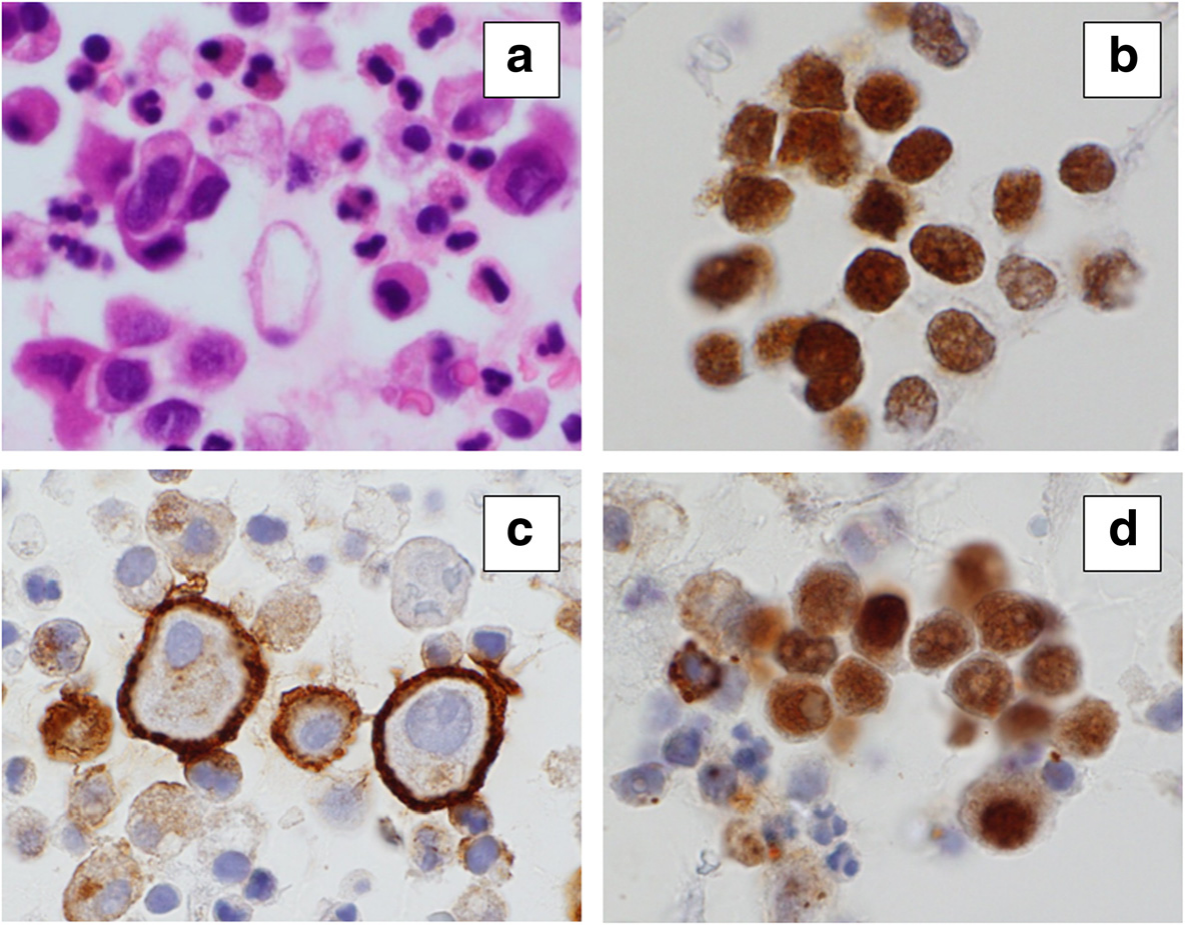
Pathological findings from the cell-block specimen of pericardial effusion. Atypical large mesothelial cells had proliferated forming a tumor nest. Hematoxylin–Eosin (**a**), calretinin (**b**), D2-40 (**c**), and Wilms’ tumor 1 (WT1) (**d**) (×1000)

The carcinoembryonic antigen (CEA) level in the pericardial effusion was 3.3 ng/mL. Pathological examination of the cell-block specimen obtained from the pericardial effusion revealed malignant mesothelial cells (Fig. 2a), which were positively stained with calretinin, D2-40, and Wilms’ tumor 1 (WT1) (Fig. 2b–d, respectively). Furthermore, the cells were positively stained for p53 and epithelial membrane antigen and negatively stained for CEA (data not shown). Thus, he was diagnosed with PPM. Considering his age, he did not receive any chemotherapy. To date, only a loop diuretic has been administered for controlling the pericardial effusion. He was discharged on the 20th day of hospitalization. Chest CT 8 (Fig. 1c) and 16 months (Fig. 1d) later revealed that the pericardial effusion was well controlled. Although his symptoms of general fatigue and trunk pain slowly but steadily progressed and the pericardial effusion slightly increased, he remained fully active 18 months after his initial presentation.

### Discussion

The treatments for PPM are surgical therapy, radiotherapy, and chemotherapy. Most of the surgical therapy comprises pericardial resection even when the tumor is localized. It is mostly useful for preventing cardiac tamponade or for tumor reduction [4]. Systemic chemotherapy with the combination of platinum and pemetrexed or the combination of doxorubicin, vincristine, and cyclophosphamide has been found to be effective for PPM in some reports [5–7]. The condition is usually fatal within a year regardless of its treatment being successful [3]. To the best of our knowledge, PPM is a rare disease entity and no reliable clinical trials for systemic chemotherapy have been conducted. The effectiveness of systemic chemotherapy had been demonstrated mainly through case reports. Because no large-scale clinical randomized trial of chemotherapy or radiotherapy for PPM has been performed yet, the evidence level for these treatments is rather low. Therefore, although neither radiation therapy nor chemotherapy has proven to be beneficial, both have been used as adjuvant treatments in patients with incomplete tumor resection or metastatic disease. In the present case, because of the patient’s age and the risk of adverse events caused by chemotherapy, we performed best supportive care alone. The patient remained fully active 18 months after his initial presentation.

The cell-block specimen obtained from his pericardial effusion was positively stained for calretinin, D2-40, and WT1 and negatively stained for CEA. Because mesothelioma cells do not have a specific marker for diagnosis, it is very difficult to make an adequate cytological diagnosis among the types of pulmonary adenocarcinoma cells. In general, useful diagnostic mesothelial markers include calretinin, WT1, cytokeratin 5/6, and D2-40. It has been recommended that at least two mesothelial markers and two carcinoma markers with greater than 80 % sensitivity and specificity be used for the diagnosis of mesothelioma when all clinical, radiologic, and histologic features are concordant [8].

These findings indicated that the pericardial tumor cells of this patient resembled epithelial mesothelioma (EM). EM generally has a good prognosis compared to sarcomatoid and biphasic mesothelioma. In particular, well-differentiated papillary mesothelioma, a rare variant of EM, is associated with a good prognosis owing to its clinically indolent behavior and long survival period [9]. Although definite reasons for the long survival time were not clear, we speculated that this favorable outcome could be obtained because of the properties of the tumor in this case.

The cause of PPM remains unknown, unlike that of pleural mesothelioma. Although the pathogenesis of PPM may not be related to asbestos exposure, Warren et al. reported that PPM was likely to develop during childhood and among patients with low levels of exposure to asbestos [3]. Mensi et al. reported a high incidence of occupational asbestos exposure among seven patients with PPM (5/7 patients: 71.4 %, 95 % CI 29.0–96.3) [10]. The asbestos exposure may be one of the main causes of PPM. Although PPM generally occurs in young people (median age, 46 years; range, 19–76) compared to pleural or peritoneal mesothelioma [11, 12], our case was an aged patient. Although his occupational asbestos exposure had lasted for only 2 years when he was young, pleural plaques detected on chest CT suggested that his disease was related to asbestos exposure.

## Conclusions

In conclusion, this aged patient with PPM experienced an indolent clinical course with a long-term palliation after pericardiocentesis and loop diuretics. Because PPM is very rare, reporting the concise course of the disease will contribute to the accumulation of clinical knowledge.

## Consent

Written informed consent was obtained from the patient for publication of this case report and accompanying images. A copy of the written consent is available for review by the Editor-in-Chief of this journal.

## Additional file

Additional file 1: Figure S1. Detailed clinical course of the pleural plaques detected in this case. Four chest computed tomographies (CTs) at different time points revealed pleural plaques. A year before disease onset (A), at the time of disease onset (B), 8 months after disease onset (C), and 16 months after disease onset (D). (TIFF 708 kb)

## Abbreviations

CEA: carcinoembryonic antigen
CT: computed tomography
PPM: primary pericardial mesothelioma
WT1: Wilms’ tumor 1
