# Supplementary figures and images for: Successful palliation for an aged patient with primary pericardial mesothelioma
Source: World J Surg Oncol. 2015 Sep 17;13:273. doi: 10.1186/s12957-015-0692-5 (PMC4573695; doi:10.1186/s12957-015-0692-5)

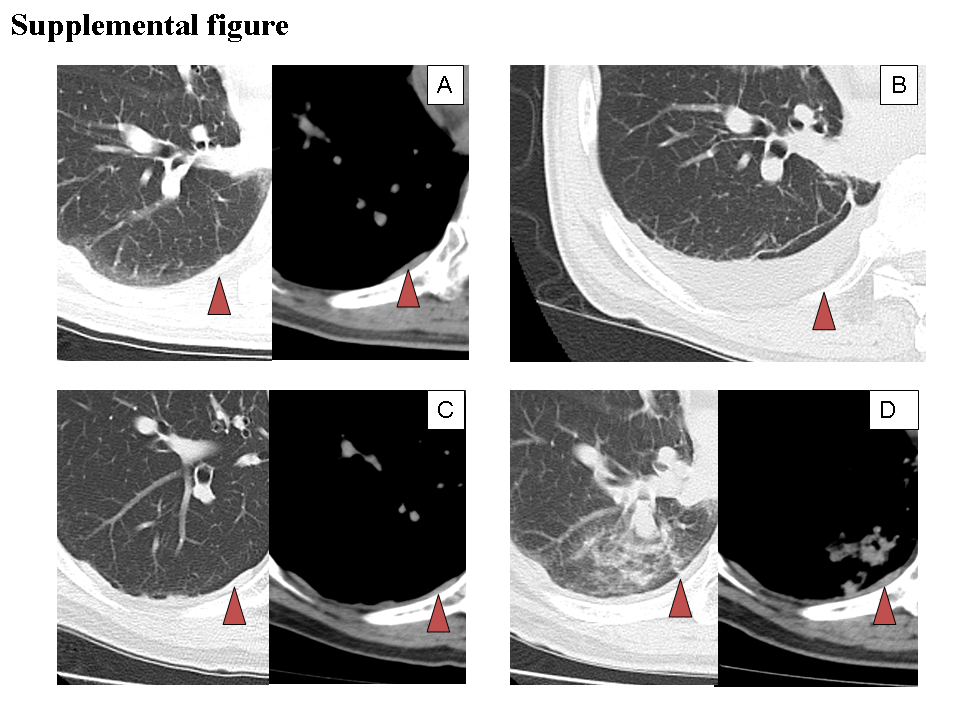

Supplement: Additional file 1: Figure S1. — Detailed clinical course of the pleural plaques detected in this case. Four chest computed tomographies (CTs) at different time points revealed pleural plaques. A year before disease onset (A), at the time of disease onset (B), 8 months after disease onset (C), and 16 months after disease onset (D). (TIFF 708 kb) [file 12957_2015_692_MOESM1_ESM.tif]
